# A Study of Second-Phase Precipitates and Dispersoid Particles in 2024 Aluminum Alloy after Different Aging Treatments

**DOI:** 10.3390/ma12244168

**Published:** 2019-12-12

**Authors:** Anna Staszczyk, Jacek Sawicki, Boguslawa Adamczyk-Cieslak

**Affiliations:** 1 Institute of Materials Science and Engineering, Lodz University of Technology, Stefanowskiego 1/15, 90-924 Lodz, Poland; anna.staszczyk@edu.p.lodz.pl; 2 Faculty of Materials Science and Engineering, Warsaw University of Technology, Woloska 141, 02-507 Warsaw, Poland; boguslawa.cieslak@pw.edu.pl

**Keywords:** precipitation hardening, microstructure, artificial aging, 2024, aluminum alloys

## Abstract

Aluminum alloys such as AA2024 are popular in the automotive and aircraft industries. The application of artificial aging significantly improves their mechanical properties by precipitation hardening. However, commercial alloys very often contain different amounts of elements such as Si and Fe that make the evolution of the microstructure harder to control. Large intermetallic particles can influence the overall results of heat treatment and cause deterioration of material properties. The authors decided to examine changes in the microstructure of three commercial 2024 alloys with varying chemical compositions by applying three different types of aging treatments. The results show considerable differences in the amount, size and morphologies of the precipitates. Second-phase Al_2_Cu and Al_2_CuMg precipitates were identified in one of the alloys. Other interesting types of multiphase particles were discovered in alloys with higher Si contents. The results show that even small variations in the composition can lead to a completely different microstructure.

## 1. Introduction

The 2024 aluminum alloy has been commonly used in the aircraft industry for many years. The most common and well-understood process of improving its mechanical properties is the artificial aging treatment (T6) at high temperature. It results in precipitation hardening following a sequence of forming S”, S’ and S (Al_2_Cu) phases from the super-saturated solid solution [1,2].

The phenomenon of secondary aging was observed in Al–Zn alloys by Löffler et al. [3]. It was observed that when aging was interrupted before reaching its peak, further natural hardening of the material can lead to obtaining even higher hardness and strength of the alloy. Secondary precipitation during interrupted treatments was thoroughly investigated by Lumley et al. [4,5,6]. It was suggested that the interruption period at a low temperature followed by another stage of aging at an elevated temperature results in finer dispersion of precipitates in the alloy [7].

It has been observed that hardening in 2024 Al alloy occurs in two stages separated by a plateau. The first, rapid increase of hardness takes place in less than 10 min when heated to a high temperature [8]. In terms of interrupted treatment of Al–Cu–Mg alloys, it is believed that the dwell period in room temperature allows more Cu–Mg clusters to nucleate at the nanometric scale of the alloy, as in the study by Marceau et al. [9]. According to the up-to-date literature studies, clusters of less than 5 nm contribute significantly to the strengthening effect; sometimes to a greater extent than S and S’ particles (S’ phase is sometimes associated with the Guinier-Preston-Bagaryatsky zones in Al alloys contrary to the previous belief that S phase, with its metastable variants, nucleates on the GPB zones [10]). However, Risanti et al. applied interrupted aging on 2024 alloy and obtained no improvement in comparison to T6 treatment [11]. Conflicting results published by researchers regarding the hardening effect of 2024 alloy may be attributed to the variations in chemical composition.

Silicon and iron have poor solubility in the alpha solution of an Al–Cu–Mg alloy. They form intermetallic phases with copper, magnesium and manganese during solidification of the cast. It is not possible to dissolve them entirely during solution treatment. Formation of those large dispersoid particles also extracts Cu and Mg from the supersaturated solution, decreasing the number of elements that could form clusters or particles during the strengthening phase. Mrówka-Nowotnik and Sieniawski examined precipitates of phases present in the microstructure just after casting [12]. The intermetallic particles were much harder and more brittle than the matrix or S phase, as measured by Radutoiu et al. [13]. It was discovered, e.g., in work by Boag et al. [14], that many of them were, in fact, multiphase with heterogeneous compositions. There is very little knowledge about mechanisms responsible for the formation of multiphase precipitates. Kaczmarek et al. optimized the process of heat treatment of 2024 and 7075 alloys so that they obtained particles of unique core–shell morphology [15]. This was followed by the latest study, done by Lipa et al., stating that those precipitates significantly improved the fatigue behavour of the 2024 alloy [16].

The research performed by Campestrini et al. demonstrated that intermetallic particles, especially of core–shell morphology, could contribute to pitting corrosion in the material [17]. They found out that the surface potential difference between the core and shell of the particle is higher than that typically found between precipitates and the matrix phase of the alloy. The standard assumes that the amount of silicon in 2024 alloy can vary from 0% to 0.5%. It has been proven that the amount of Si can strongly influence the precipitation process in Al alloys [18,19,20].

The presence of large brittle particles of intermetallic phases can lead to deterioration of mechanical properties as they serve as crack initiation points. This decrease in toughness is a serious issue faced when applying aluminum alloys for construction applications. Therefore, it is important to investigate this problem and find ways to avoid it by altering the microstructure of the material through alloying elements or heat treatment conditions. So far, not enough research has focused on finding differences in the behavior of 2024 alloy with varying amounts of alloying elements. From an industrial point of view, it is not possible to eliminate Si and Fe impurities from high-strength Al alloys. The purpose of this research was to prove that, in commercial 2024 alloy with composition in the range described by a standard, there might be considerable differences in the obtained microstructures and therefore predicted hardening response.

## 2. Materials and Methods

Three different 2024 alloys with varying chemical compositions were studied, denoted as A, B and C. Their compositions are shown in Table 1. Each of the alloys was bought from a different supplier as a standard AA2024 and their chemical composition was examined using an ARL Perform’X™ Sequential WDXRF X-ray spectrometer.

**Figure 1:**
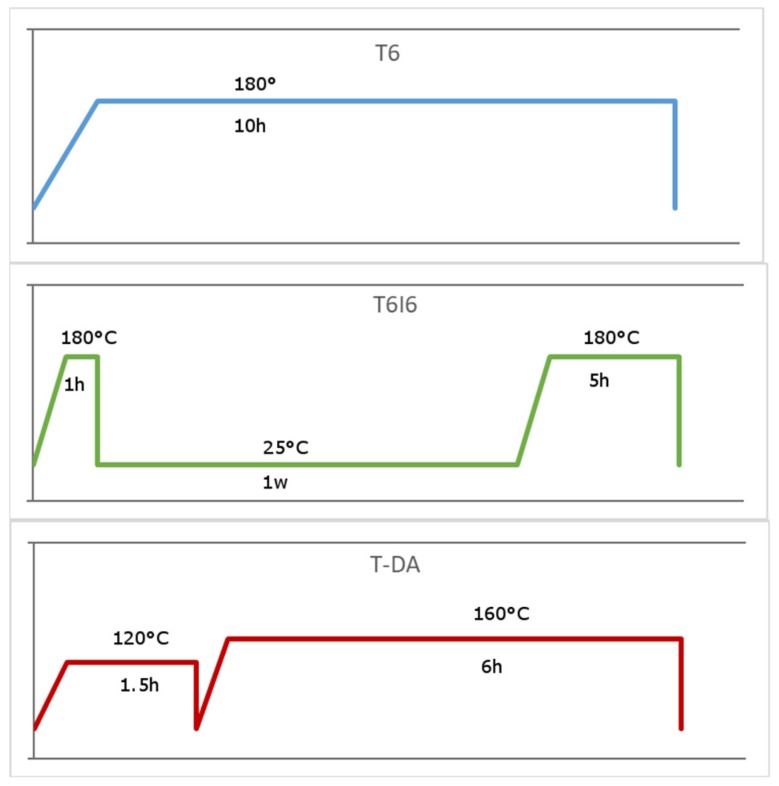
Schematic representation of three different aging treatments applied to the examined alloys.

All of the samples were solution treated at 500 °C for 4 h, then quenched in room temperature water. This process was optimized during preliminary research. Parameters of aging applied to the alloys were developed based on common industry standards and literature studies. T6 aging was performed for 10 h at 180 °C, which is a widely applied practice for 2024 alloy. T6I6 aging treatment was performed at the same temperature, but in two stages (1 and 5 h) interrupted by a week at room temperature. Finally, the innovative T-DA (double aging) treatment was introduced based on the good results obtained by Kaczmarek et al. [21]. The schematic representation of the processes is shown in Figure 1. The Vickers hardness of specimens was measured after solution treatment and then after each following process. The measurements were performed on the Innovatest Verzus 700AS tester (INNOVATEST Europe BV, Maastricht, The Netherlands) with a 5 kg load.

All of the specimens after heat treatment were prepared for scanning electron microscopy (SEM) observation with traditional methods; i.e., ground with papers from 300 to 2400 grade and polished with 0.03 µm colloidal silica. When not under examination, the specimens were stored in a freezer at −18 °C to avoid natural aging processes. 

Scanning electron microscopy observations were carried out on the JEOL JSM-6610LV equipment (Jeol Ltd., Tokyo, Japan). The 20 keV accelerating voltage was applied with a 10 mm working distance.

SEM micrographs were taken of at least ten randomly chosen regions of each specimen with magnification 1000× to determine the amount of secondary phases in the microstructure. Energy dispersive spectroscopy (EDS) was used to determine chemical compositions of selected phases. 

These pictures were then analyzed with image processing software to calculate a number of precipitates and the areas occupied by them. The obtained results were then analyzed to compare the effects of different compositions and treatments.

## 3. Results and Discussion

The results of the Vickers hardness measurements of the specimens are shown with the calculated standard deviation in Table 2. There is a tendency that alloy A has the highest hardness of all after every treatment. Also, it obtained peak hardness after the T-DA treatment and had the lowest value after T6I6. For the rest of the alloys, there were no differences in hardness between aging types. It may be explained by comparing the volume fractions of intermetallic phases present in the microstructures which are on the same level for all specimens of alloys B and C.

**Figure 2:**
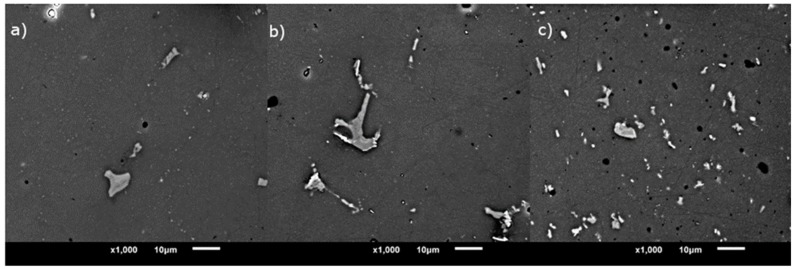
Comparison of microstructures of (**a**) alloy A, (**b**) alloy B, and (**c**) alloy C after T6 aging.

**Figure 3:**
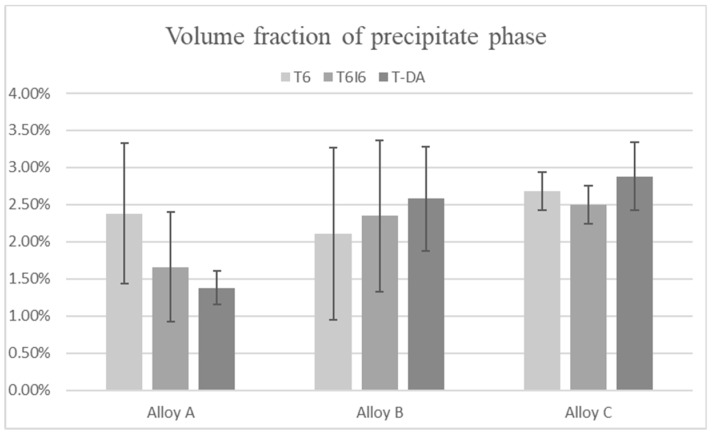
Total volume fractions of precipitate phases for all the alloy treatment variants.

There is a considerable difference between the microstructures of alloys with different chemical compositions, and the tendency remains the same for every heat treatment type. The comparison of microstructures at the same magnification is displayed in Figure 2. The fraction of the precipitate phase is shown in Figure 3, and it was calculated from micrographs as the amount of area occupied by precipitates in relation to total area of an image. For alloy A, there is a small drop in the amount of precipitate phase after combined treatments T6I6 and T-DA, in contrast to T6. Also, the lowest volume fraction corresponds to the peak hardness of the alloy. For alloys B and C there is no such tendency; there is even a noticeable increase in volume fraction, especially for T-DA treatment. Overall, the differences between the variants are not significant and the values are all in the range of standard deviation.

**Figure 4:**
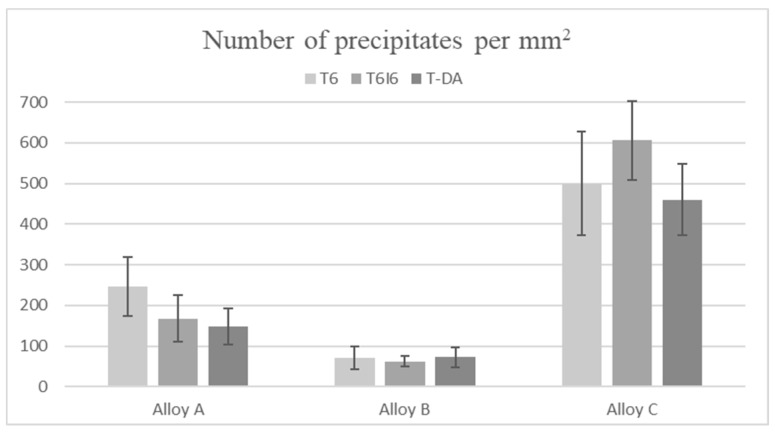
Number of precipitates on the examined surface of the specimen per mm^2^.

**Figure 5:**
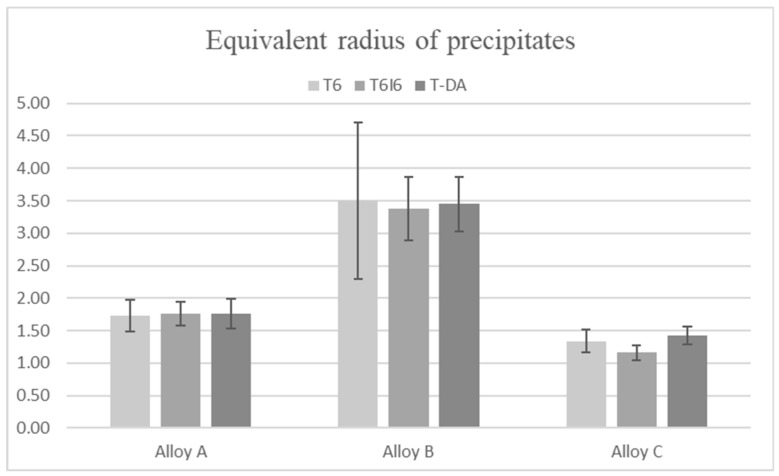
The equivalent radius of precipitates on the examined surface of the specimen.

The calculated number of precipitates per 1 mm^2^ is shown in Figure 4. In the case of this parameter, there is a very distinct difference between the alloys. The microstructure of alloy B has much fewer particles than the other two. However, the most drastic difference is visible for alloy C, where there are up to 5 times more precipitates than in alloys A and B. Those results are firmly connected with the mean radii of particles shown in Figure 5. It was calculated as an equivalent radius of an average round precipitate resulting from the area occupied by it. In alloy B, with the lowest number of particles per unit of area, the precipitates are the largest, whereas in alloy C, the precipitates are numerous but very small.

**Figure 6:**
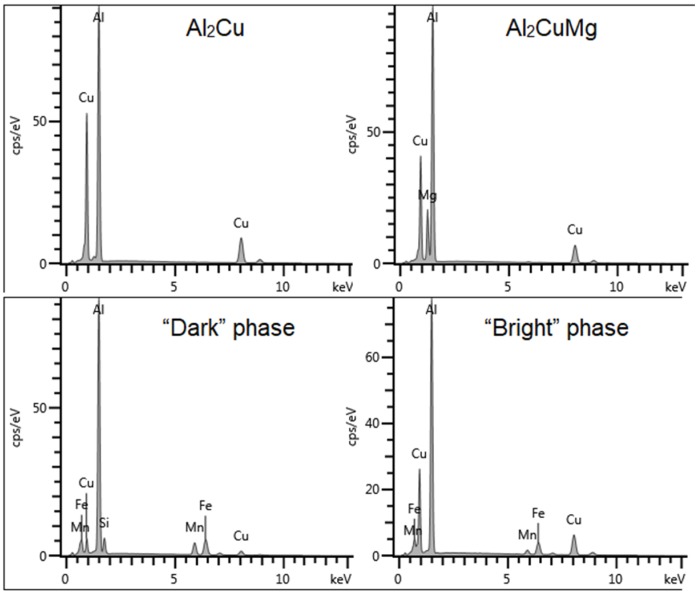
Spectra from EDS analysis of the most common phases found in the alloys A, B and C.

**Figure 7:**
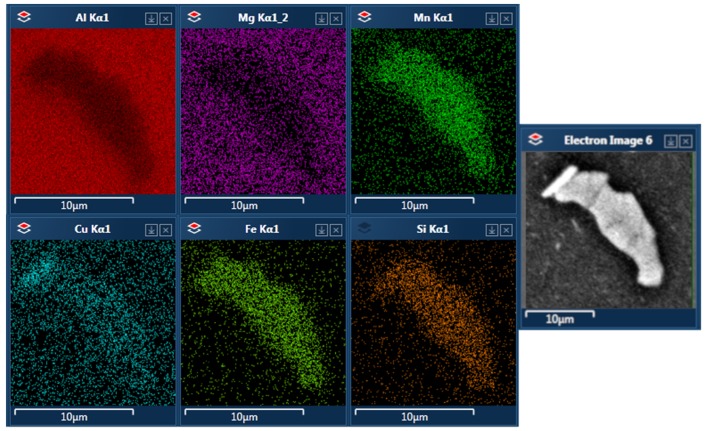
A precipitate found in the alloy A after T-DA aging.

**Figure 8:**
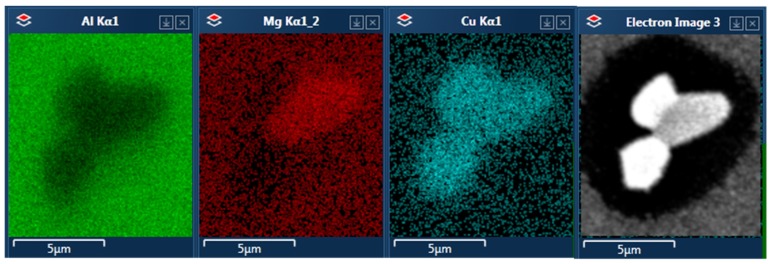
Precipitates of Al_2_Cu and Al_2_CuMg phases found in alloy A.

Single precipitates were analyzed with EDS to determine their chemical composition. The element weight % contents of the most representative phases are shown in Table 3. The corresponding spectra are shown in Figure 6. In alloy A, two different types of particles were observed. The first were larger particles with irregular shapes consisting of Al, Cu, Mn, Fe and Si, as shown in Figure 7. There is no differentiation in chemical composition within the precipitate. There is also a second type of particle present, close to round in shape and visually much brighter, in contrast to the others on SEM images. Precipitates of this type contain Al and Cu with a weight ratio of 60%:40%, and some of them also have Mg in smaller amounts (Figure 8). Based on the EDS results, and on their visual characteristics, they were identified as Al_2_Cu and Al_2_CuMg, which corresponds to the well-known θ and S phases, respectively. Despite the fact that they are usually observed with nanometric sizes, it is not unheard of to find larger particles of this type, as reported by, e.g., Buchheit et al. [22]. Such precipitates were not commonly observed in other alloys, where higher contents of Si and Fe might have encouraged the formation of more complex phases instead.

**Figure 9:**
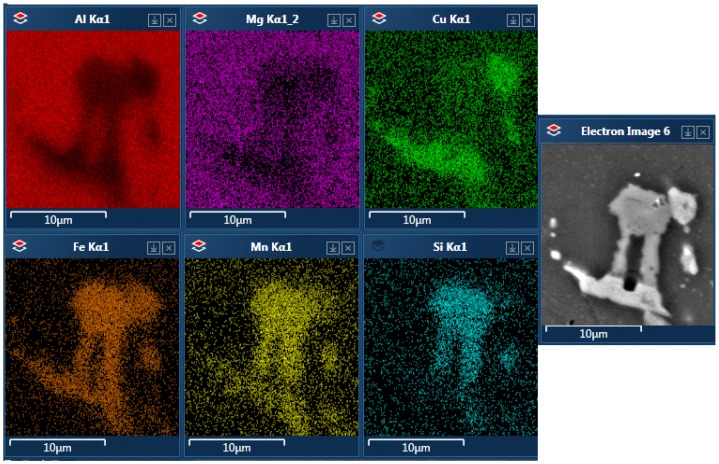
A precipitate found in alloy B after T-DA aging.

Particles of intermetallic phases in alloy B are visibly larger and more elongated in shape than in the two other alloys. They often form complicated and curved structures with ‘empty’ places in the middle of their cross-section. Their chemical composition is very close to the bigger phases observed in alloy A, as EDS results in Figure 9 show. There were virtually no θ and S precipitates visible.

In alloy B, multiphase precipitates were observed, such as in Figure 9. One phase had more Cu and another had more Si. The Cu-rich phase appeared brighter in the image and was very often located near the edges of precipitates. For identification purposes, it is referred to as the “bright” phase, while the phase with lower Cu content was called the “dark” phase. The EDS results for both can be found in Table 3 and in Figure 6.

**Figure 10:**
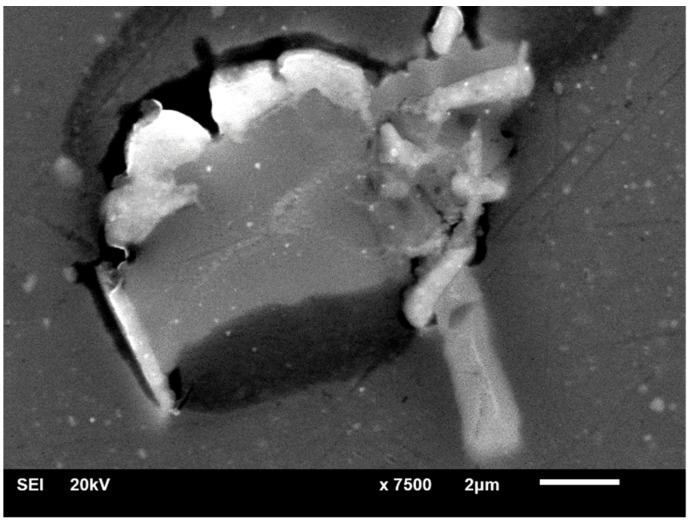
A closer view at the multiphase precipitate found in alloy B after T6I6 aging showing the distinction between “bright” and “dark” phases.

A fragment of the precipitate showing the boundary between the two phases is presented in greater magnification in Figure 10. This phenomenon was never observed in alloy A, which leads to the conclusion that this specific morphology of precipitates is correlated with higher Si and Fe contents in the alloy.

**Figure 11:**
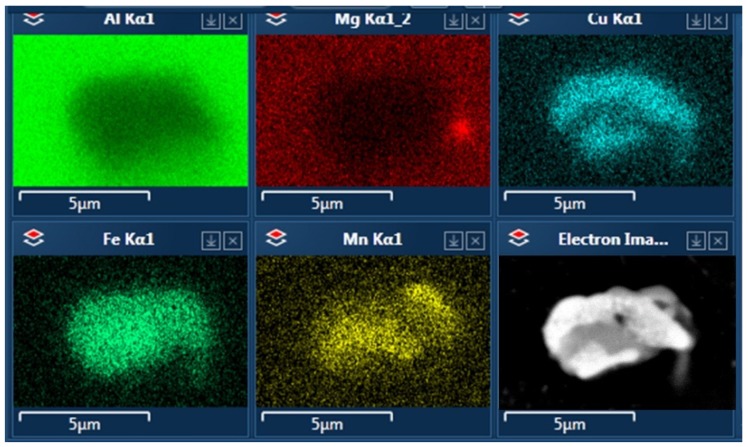
A precipitate of core–shell morphology found in alloy C after T6 aging. The results of EDS analysis show differentiation of chemical composition between the two phases.

A similar diversity of phases was observed in alloy C. An interesting type of precipitate morphology was observed in this alloy, where one particle consists of two phases merged in a form resembling a core and shell. The results of the EDS analysis of these particles are shown in Figure 11. Here, again, the brighter phase is rich in copper and it tends to occur on the circumference of the precipitate.

In the microstructure of alloy C, there is a much finer dispersion of particles; they are considerably smaller and densely distributed. Again, no distinguishable θ and S phases were observed. However, all of the particles tended to be more regular and rounder than in the two other alloys. The microstructure of this alloy matches the description of possible core–shell particles described in previous studies of 2024 alloy, though it was not possible to find a connection between their presence and the parameters of heat treatment. Similar looking particles with the same compositions were found in specimens according to each aging type, but they did not influence the hardness of the alloy. 

## 4. Conclusions

1. There was a considerable difference in the microstructure of alloys containing varying amounts of added Si and Fe. The total volume fraction of intermetallics remained at the same level for all of them; huge differences in size and dispersion of particles were observed. Alloy A, with small amounts of Si and Fe, contained two distinct types of particles. Alloy B, in the middle range of the standardized composition, contained phases much bigger than the other two, with curved, complicated shapes. Alloy C, in the upper limit of alloying additions, had a high number of smaller and rounder precipitates.
2. There was no significant difference in hardness after different aging treatments for one alloy. Alloy A had the highest hardness after solution treatment, and the tendency remained the same after all processes. Process parameters did not influence the characteristics of intermetallic particles in the material.
3. Alloy A, with the lowest additions of Si and Fe, was the only one to have numerous Al_2_Cu and Al_2_CuMg precipitates. This leads to the conclusion that higher Si and Fe contents prevent the θ and S phases from growth on a microscopic scale.
4. In alloys B and C, with higher Si and Fe contents, the presence of multiphase precipitates was confirmed, as predicted by literature study. The difference between chemical compositions of those phases was most prominent in Cu contents.
5. In alloy C, some of the multiphase precipitates showed core–shell-like morphology. It looks like their presence correlated to higher Si and Fe content, but not heat treatment type. We conclude that the beneficial effects connected with multiphase aging treatments could be caused by the specific chemical composition of the 2024 alloy used in a study.

## Figures and Tables

**Table 1:** Chemical composition of three examined 2024 alloys in % mass.

Alloy	Cu	Mg	Mn	Si	Fe	Zn	Cr	Ti	Ni	Al
A	4.76	1.36	0.79	0.16	0.12	0.04	0.02	0.02	0.01	Rest
B	4.15	1.51	0.64	0.24	0.25	0.09	0.01	0.06	0.01	Rest
C	4.39	1.62	0.74	0.41	0.43	0.04	0.01	0.05	0.01	Rest

**Table 2:** Values of measured Vickers hardness of examined alloys after heat treatment.

Alloy	T	T6	T6I6	T-DA
A	110 ± 2.6 HV	138 ± 2.7 HV	135 ± 1.3 HV	143 ± 2.9 HV
B	102 ± 2.6 HV	134 ± 1.9 HV	132 ± 3.1 HV	133 ± 1.0 HV
C	105 ± 3.2 HV	135 ± 3.2 HV	132 ± 2.6 HV	133 ± 2.4 HV

**Table 3:** Chemical compositions of the observed phases obtained by EDS. The corresponding spectra are shown in Figure 6.

Element	wt % in Phases			
	Al_2_Cu	Al_2_CuMg	“Bright” Phase	“Dark” Phase
Al	60.75 ± 0.25	60.72 ± 0.20	57.36 ± 0.28	59.84 ± 0.25
Cu	39.25 ± 0.25	28.40 ± 0.21	30.38 ± 0.29	6.20 ± 0.13
Mg		10.89 ± 0.10		
Mn			2.89 ± 0.11	10.75 ± 0.17
Fe			9.36 ± 0.17	15.53 ± 0.20
Si				7.69 ± 0.13
